# Leveraging Citizen Science for Healthier Food Environments: A Pilot Study to Evaluate Corner Stores in Camden, New Jersey

**DOI:** 10.3389/fpubh.2018.00089

**Published:** 2018-03-26

**Authors:** Benjamin W. Chrisinger, Ana Ramos, Fred Shaykis, Tanya Martinez, Ann W. Banchoff, Sandra J. Winter, Abby C. King

**Affiliations:** ^1^Stanford Prevention Research Center, Stanford University School of Medicine, Palo Alto, CA, United States; ^2^The Food Trust, Philadelphia, PA, United States; ^3^Department of Health Research and Policy, Stanford University School of Medicine, Stanford, CA, United States

**Keywords:** citizen science, community engagement, food access, food deserts, healthy corner stores, empowerment, program evaluation

## Abstract

Over the last 6 years, a coordinated “healthy corner store” network has helped an increasing number of local storeowners stock healthy, affordable foods in Camden, New Jersey, a city with high rates of poverty and unemployment, and where most residents have little or no access to large food retailers. The initiative’s funders and stakeholders wanted to directly engage Camden residents in evaluating this effort to increase healthy food access. In a departure from traditional survey- or focus group-based evaluations, we used an evidence-based community-engaged citizen science research model (called *Our Voice*) that has been deployed in a variety of neighborhood settings to assess how different features of the built environment both affect community health and wellbeing, and empower participants to create change. Employing the *Our Voice* model, participants documented neighborhood features in and around Camden corner stores through geo-located photos and audio narratives. Eight adult participants who lived and/or worked in a predefined neighborhood of Camden were recruited by convenience sample and visited two corner stores participating in the healthy corner store initiative (one highly-engaged in the initiative and the other less-engaged), as well as an optional third corner store of their choosing. Facilitators then helped participants use their collected data (in total, 134 images and 96 audio recordings) to identify and prioritize issues as a group, and brainstorm and advocate for potential solutions. Three priority themes were selected by participants from the full theme list (*n* = 9) based on perceived importance and feasibility: healthy product selection and display, store environment, and store outdoor appearance and cleanliness. Participants devised and presented a set of action steps to community leaders, and stakeholders have begun to incorporate these ideas into plans for the future of the healthy corner store network. Key elements of healthy corner stores were identified as positive, and other priorities, such as improvements to safety, exterior facades, and physical accessibility, may find common ground with other community development initiatives in Camden. Ultimately, this pilot study demonstrated the potential of citizen science to provide a systematic and data-driven process for public health stakeholders to authentically engage community residents in program evaluation.

## Introduction

### Background/Rationale

Camden, New Jersey is a former industrial city of approximately 74,000 residents located directly across the Delaware River from Philadelphia, Pennsylvania, and has struggled with extended periods of decline, high rates of poverty, and unemployment (for a map of Camden, see Figure S1 in Supplementary Material) (1). Decades of challenging economic conditions coupled with major shifts in the food retailing industry have left most of Camden’s residents with little or no access to large supermarkets. Though Camden is largely considered to be a “food desert,” an array of approximately 120 smaller corner or convenience food stores are embedded in the city’s neighborhoods, providing one possible avenue for improving physical access to healthy foods (2). However, as previous research has shown, these smaller retail formats typically lack healthy food options, especially fresh fruits and vegetables, which are affordable, high quality, and acceptable to local residents (3–5).

Over the last 6 years, a coordinated Camden Healthy Corner Store Network has helped an increasing number of local storeowners stock, market, and sell healthy, affordable foods (6–8). This program is modeled, in part, on a similar and widely emulated initiative in nearby Philadelphia, PA that also sought to increase food access in urban neighborhoods by focusing on existing small, neighborhood retailers that often lack fresh or healthy food options (4). At the time of this study’s initiation in 2016, 35% of Camden’s 125 corner stores were participants in the Healthy Corner Store Network (7). Participating retailers are eligible for technical assistance and equipment, like refrigerators, to carry fresh foods, as well as a range of marketing materials that encourage healthier eating (e.g., “no sodium added canned beans”) and moderation or avoidance of less-healthy options (e.g., high-sodium products) (6). Ten retailers in the Healthy Corner Store Network are also part of a broader nutrition education program that allows participating Camden residents to redeem “Heart Bucks” vouchers for healthy foods at their stores. The coordinators of this program, a food access advocacy nonprofit, The Food Trust (TFT), were interested in engaging residents to conduct a formative evaluation of current efforts and maximize the potential for community benefit through the program (9).

### Healthy Corner Store Evaluations

Evaluations of the Philadelphia healthy corner store initiative and others like it in the United States and abroad have documented significant positive changes in the availability of healthy food in targeted neighborhoods, but have found limited evidence for these interventions’ effect on individual-level purchasing or dietary patterns (10–13). Most healthy corner store evaluations typically feature audits of the in-store and surrounding environments, though some also include perspectives of community residents through shopper intercept surveys (14). These evaluations often replicate best practices of measuring and monitoring the retail food environment by employing validated surveys such as the Nutrition Environment Measures Survey or follow international expert data collection guidelines, such as those published by the International Network for Food and Obesity/non-communicable diseases Research, Monitoring and Action Support (10, 15, 16). While critical for quantification of changes in the retail environment, these tools inherently limit possible community input; when residents are engaged to solicit their perspectives or opinions, they often respond to predefined questions, or must recall previous experiences rather than directly react to what is contextually before them.

Alternatively, customer focus groups or interviews provide participants broader latitude to express their perceptions, and some studies have also incorporated feedback from participating store owners into their evaluations of healthy corner store programs (17). However, when interviews or focus groups take place outside of the environment where food shopping takes place, in contrast to more intensive methodologies such as walking- or go-along interviewing (18, 19), more detailed observations of reactions or responses are not possible.

### Photovoice

Photovoice is one example of a participatory qualitative methodology that has been used in public health evaluation research. Previous studies have used Photovoice in participatory evaluations of community-based obesity prevention programs, as well as school-based gardening programs (20, 21). Additionally, Photovoice projects have been used to examine public policy issues or arenas for potential intervention. For instance, the Witnesses to Hunger program uses Photovoice methods to engage low-income participants in informing policymakers about the role of social welfare in their lives, including the Supplemental Nutrition Assistance Program (SNAP, formerly known as Food Stamps) (22). Other Photovoice projects have documented food shopping trips among low-income women in food deserts (23), environmental influences on eating behaviors (24), as well as the daily experiences of homeless individuals (25). Some studies have noted the potential use of Photovoice as a participatory action tool, with potential advantages to participants, even those with limited political power (26).

The community-engaged research model used in this study, called *Our Voice*, extends the strengths of Photovoice and other “citizen science” observational methods by introducing a data-driven, solution-oriented process to generate positive health-related changes in local environments (27). The *Our Voice* model has been used previously to assess a variety of neighborhood environment issues, including healthy food access among seniors in the San Francisco Bay Area, and has been shown to mobilize changes at individual and community levels (27, 28). This investigation represents the first use of *Our Voice* as part of a formative evaluation of a public health intervention.

The purpose of this pilot study was twofold: (1) document Camden resident perceptions of the Camden Healthy Corner Store Network’s efforts, as well as their ideas for the future and (2) explore the viability of using the *Our Voice* citizen science model for evaluating an existing food environment intervention.

## Methods

### Participant Recruitment

As in other projects that use the *Our Voice* research model [described in detail by King et al. (27)], the Stanford University team used a train-the-trainer approach with TFT staff who were already working on Camden initiatives. TFT staff then recruited and consented (*via* written consent form) adult participants by convenience sample, identified corner stores to participate as study sites, and scheduled and supervised participant engagement. Interested individuals were eligible to participate if they lived or worked in a specific North Camden neighborhood, were at least 18 years old, could complete written forms in English or Spanish, and were physically able to complete a walk of approximately 20 min. Following the first part of the study (neighborhood walk), participants were offered a $25 gift card. Participants were offered a second $25 gift card following the second part of the study (Community Meeting 1). The Institutional Review Board at Stanford University approved this study in August 2016.

### Corner Store Assessment Walks

Upon arriving at their appointed time/location, participants were trained (approximately 10 min) to use a mobile application, called the Discovery Tool (loaded onto Samsung Galaxy electronic tablets) to document corner store environmental features with geo-referenced photographs and audio narratives (29). All participants were asked to visit and assess two food retailers identified in advance by TFT staff. To be selected for consideration as a store for visits, retailers had to be participating in the Camden Healthy Corner Store Network and willing to allow participants to use the DT app inside the store. TFT ultimately selected a store (Store A) that was less involved in the Network than the other store that was chosen (Store B). Participants also had an option to assess a third store of their choosing.

Before they began their walk, participants were prompted to capture elements of each store that either supported or hindered their ability to lead healthy, active lives, and were also allowed to determine the route taken in between stops at specified retailers.

### Participant Survey

Following the neighborhood walk, participants returned their electronic tablet and completed a 49-item paper survey consisting of demographic and perceived environment questions as well as information about food shopping preferences and behaviors. Survey data were not shared with participants, but were later aggregated by researchers to help understand the study population within a broader context. Summary statistics were generated for all quantitative survey measures. Five-item Likert scales were collapsed to three items (e.g., “Strongly Agree” and “Agree” to “Agree”; “Strongly Disagree” and “Disagree” to “Disagree”; and “Neutral”) for summary tables.

### Discovery Tool Data Preparation

After the corner store assessments were completed in mid-September 2016, the Stanford University research team prepared participant Microsoft Word documents with verbatim transcriptions of each participant’s audio narratives paired with their respective photographs. These “data packets” were shared with TFT staff, who printed copies for participants.

### Community Meeting 1: Theme Selection and Prioritization

Approximately 2 weeks following their neighborhood assessments, participants received their data packets from TFT staff in Community Meeting 1, held in late September 2016. Researchers asked participants to review their photos and audio transcripts, and then facilitated a group discussion to identify a list of shared themes/issues and select a subset of these themes as priorities. Participants elected representatives to present their findings and priorities in a subsequent meeting (Community Meeting 2), and brainstormed possible stakeholders to invite to Community Meeting 2. In preparation for Community Meeting 2, TFT staff helped the participant representatives plan their presentations.

### Community Meeting 2: Priority Theme Presentation and Action Item Development

The list of themes and priorities from Community Meeting 1 provided the foundation for Community Meeting 2, held in mid-November 2016, where participants presented their findings to community leaders and decision-makers, and agreed on action items and responsible stakeholders to create changes in relation to corner store access and environments.

## Results

### Participant Characteristics

Eight participants who lived and/or worked in a predefined Camden neighborhood were successfully recruited. Half of the participants were female; five of eight were 18–25 years of age and the remaining three were 26–40 years of age. In terms of self-reported race/ethnicity, collected for comparison to broader neighborhood demographics, three participants were Black, three were Hispanic, and two were multiple races or did not specify race. Half of participants had received SNAP benefits and two had received Special Supplemental Nutrition Program for Women, Infants and Children within the past year. Participants’ average household size was 5.5 people (including themselves). Participants reported having one (*n* = 3) or no vehicles available at home (*n* = 5). Nearly all of the participants (*n* = 7) attended Community Meeting 1 that focused on participant data sharing and group consensus-building, and three volunteered to present their findings at Community Meeting 2.

### Participant Food Shopping Perceptions and Behaviors

All participants cited prices and item quality as important factors in selecting a store for their household grocery shopping. More than half of participants indicated that item selection (*n* = 7), sales, coupons or price promotions (*n* = 6), or items for special diets (e.g., low sodium, vegetarian, etc.) (*n* = 5) were also important factors in choosing a store, while only two participants said that prepared food items were important in their decision. Only two participants agreed that finding fresh fruits and vegetables in their neighborhood was easy, with the remainder disagreeing (*n* = 4) or reporting neutral feelings (*n* = 2).

**Figure 1:**
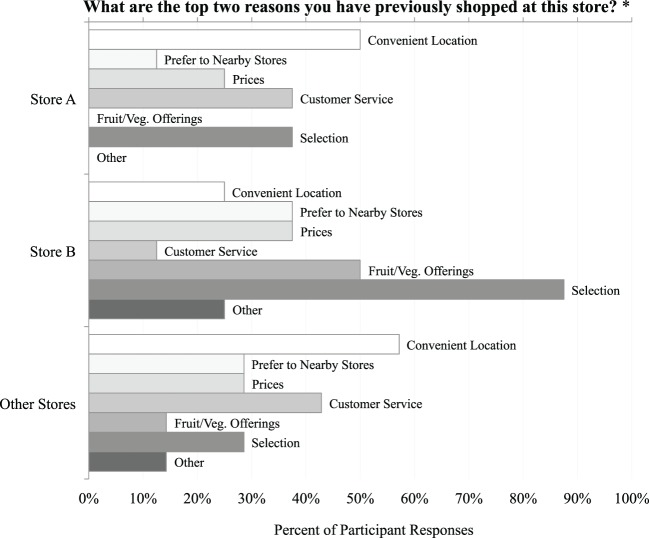
Factors for choosing a store for grocery shopping. *Some participants gave more than two reasons when responding to this question; thus, results are presented as a percent of participant responses about Store A, Store B, and Other Stores.

Most participants reported having visited both Store A (less engaged in the Healthy Corner Store Initiative) and Store B (more engaged in the Initiative) prior to completing the Discovery Tool walks; half of participants had visited Store B within the last week, while most participants (*n* = 6) had not been to Store A within the last month, and one had never been before. Of the seven participants who visited an optional third store of their choosing, three had visited the store the day before, four had visited more than one week ago, and one had never visited prior to the study. The top reasons for previously shopping at Store A were its convenient location (*n* = 4) and product selection (*n* = 3). The top reasons for shopping at Store B were fruit and vegetable offerings (*n* = 4) and product selection (*n* = 7) (see Figure 1). Participants also reported that Store B seemed to encourage healthier choices than Store A; five participants felt that Store B encouraged them to make healthy choices, while only one participant reported the same for Store A and the Other Store of their choosing.

Most participants said they would return to Store B (*n* = 6, “Yes”; *n* = 2, “Not Sure”; *n* = 0, “No”) and the Other Store of their choosing (*n* = 6, “Yes”; *n* = 1, “Not Sure”; *n* = 0, “No”), while half said the same about Store A (*n* = 4, “Yes”; *n* = 1, “Not Sure”; *n* = 3, “No”). In terms of their reasons for not wanting to return to Store A, participants selected inconvenient location, disliking the product selection, preference for other stores, and feeling unsafe/uncomfortable. When asked which of the stores visited on the walk might be incorporated into their daily lives/routines, five participants selected Store B, no participants selected Store A, two participants selected the additional store they visited, and one participant said that none of the stores visited would meet that criteria.

### Participant Neighborhood Perceptions

Participants also reported their broader perceptions about the civic, social, and built environments in their neighborhood (see Table 1). On average, participants tended toward agreement with statements about their ability to influence decisions that affect their community, both individually (mean = 3.1 out of 5, SD = 1.4) and with other community members (mean = 3.9 out of 5, SD = 1.4), and the presence of people with connections to others who can influence what happens in the community (mean = 3.9, SD = 0.9). Participants tended toward disagreement regarding statements of residents’ collective ability to solve community problems (mean = 2.6 out of 5, SD = 0.9) and the ease of buying fresh fruits and vegetables in their neighborhood (mean = 2.6 out of 5, SD = 1.7).

**Table 1:** Participant neighborhood and corner store perceptions, collected *via* self-administered paper survey, postwalk (September 2016).

Survey question	Valid *N*	Disagree	Neutral	Agree
• I can influence decisions that affect my community.	8	3	1	4
• By working together with others in my community, I can influence decisions that affect my community.	8	1	1	6
• People in my community have connections to people who can influence what happens in my community.	7	0	3	5
• If there is a problem in my community, people who live here can get it solved.	8	3	4	1
• It is easy to buy fresh fruits and vegetables in my neighborhood.	8	4	2	2
• Shopping at this store encourages me to make healthy choices.				
○ Store A	7	6	1	0
○ Store B	8	1	2	5
◦ Other Store	6	4	1	1

### Discovery Tool Data Characteristics

**Figure 2:**
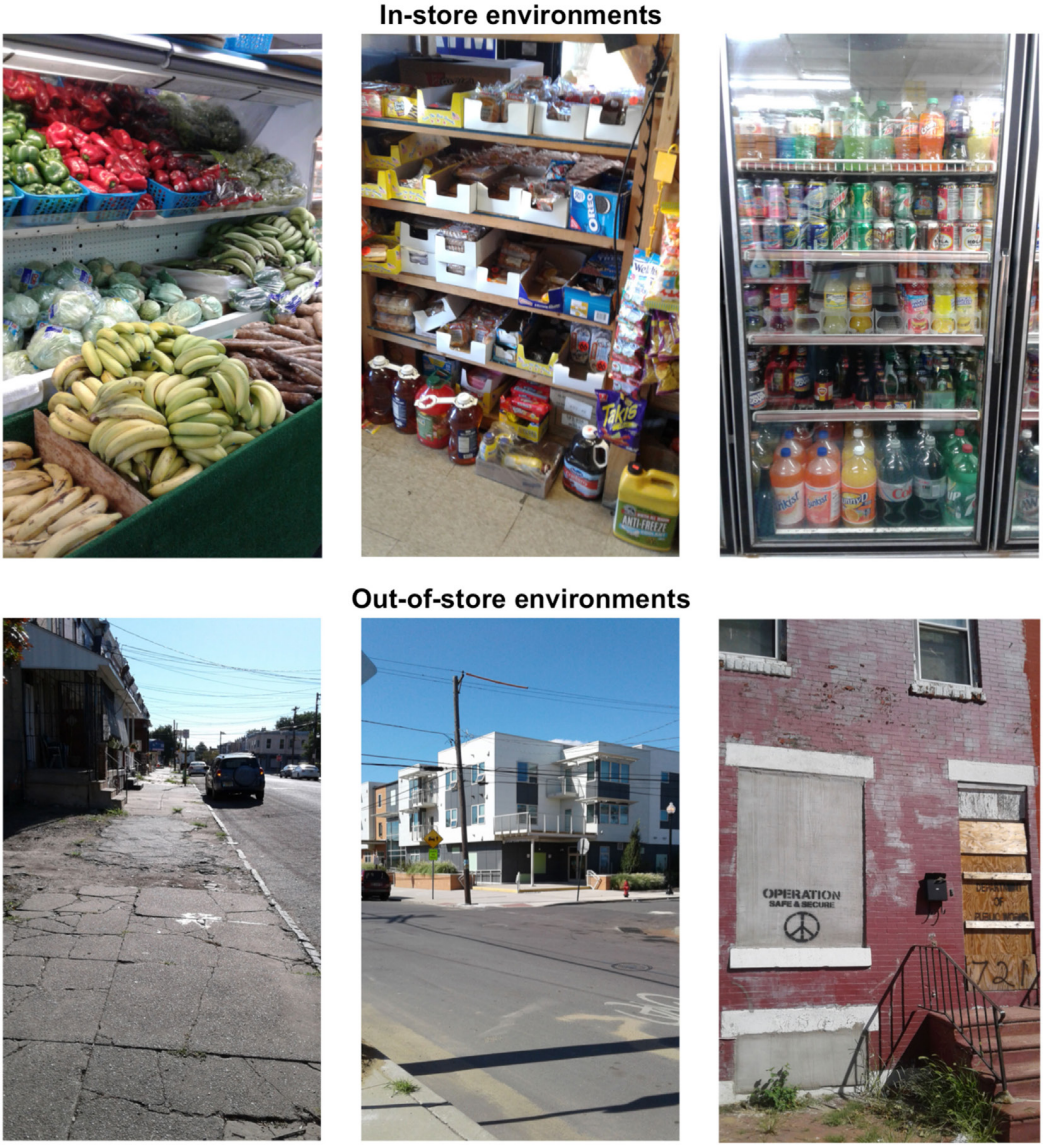
Example photographs taken by citizen scientists with the discovery tool application in Camden, New Jersey (September 2016).

In total, participants collected 134 images (mean = 16.8, SD = 10.7) and recorded 96 audio narratives (mean = 12.0, SD = 9.0) during their corner store assessments (see Figure 2, for example, photographs from inside and outside of corner stores). The majority of participants (*n* = 7) opted to visit a retailer of their choosing in addition to Stores A and B, which added data from four additional corner stores to the project.

### Themes Identified and Prioritized

**Figure 3:**
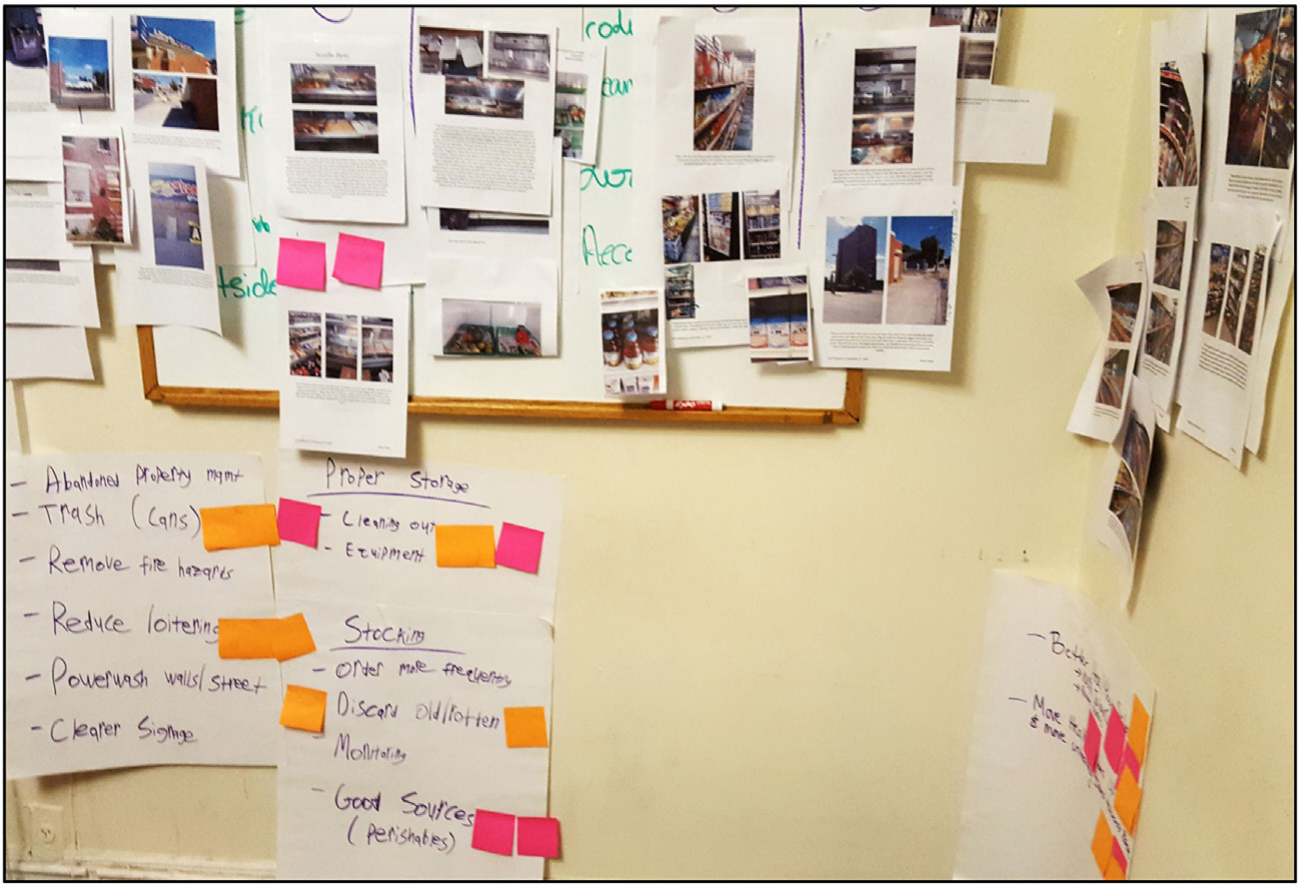
Photograph of theme clusters assembled during Community Meeting 1 in Camden, New Jersey (September 2016).

Citizen scientists identified key themes (*n* = 9) after reviewing and synthesizing their collective photo and audio narrative transcripts in the Community Meeting 1 (see Figure 3 and Table 2). These included the following: healthy product selection, store appearance and cleanliness, competition between stores, loitering and safety, walkability, freshness of food, product displays, accessibility for those with physical disabilities, and customer service.

**Table 2:** Themes identified by citizen scientists in Community Meeting 1 (September 2016) and action steps developed in Community Meeting 2 (November 2016).

Themes and priorities (in italics)	Action steps	Description
Store competition Healthy product selection Freshness of food Product displays Store appearance and cleanliness Walkability Accessibility for people with physical disabilities Loitering and safety Corner store culture	Store owner training/connections	Provide store owners with enhanced trainings on topics such as product display techniques, produce seasonality, merchandising, and marketing strategies, and connect them to produce suppliers in the city

	Additional equipment for store owners	Provide store owners with more equipment, such as baskets and shelving to increase the amount of fresh produce offered and to create a more attractive display

	Environmental improvements	Explore partnerships to support environmental improvements such as façade upgrades, security cameras installation for safety, energy audits, and others

	Continued engagement between citizen scientists and TFT	Continue to involve citizen scientists with the Camden Healthy Corner Store Network by sharing information and inviting them to relevant meetings

After group discussion of all identified themes, the citizen scientists selected the main themes (*n* = 3) to focus on based on feasibility of creating change, importance to them, and potential partners and resources. The three themes that emerged as the most important and feasible to address were related to improving healthy product selection and display, in-store environment, and a store’s outside appearance and cleanliness. These themes and priorities were presented by three of the citizen scientists at Community Meeting 2.

### Action Steps Developed

Following the Community Meeting 2, citizen scientists and local community stakeholders (including local community leaders, an education advocate, funders, and others) identified several action items that were used by TFT staff to adjust the strategic plan for the following year of programming in Camden (see Table 2).

Key elements of the Camden Healthy Corner Store Network were identified as positive, such as the provision of refrigeration and display equipment, though participants were not generally aware that these were part of an overarching citywide initiative. Potential areas for improvement included storeowner training and support. Other themes and priorities extended beyond “healthy” programming, and included observations about business and community dynamics. Some priorities, such as improvements to safety, exterior facades, or physical accessibility, may find common ground with existing community development initiatives in Camden, such as a broader Get Healthy Camden initiative (30). In the months following Community Meeting 2, several action items developed by the citizen scientists have been incorporated into planning documents for the future of the Camden Healthy Corner Store Network. Additionally, citizen scientists have been invited to participate in new programming in the community, such as local health fairs.

## Discussion

This pilot study allowed a group of Camden residents to critically assess and reflect on the successes and opportunities for improvement in a major community health promotion effort. Participants contrasted their perceptions of two different participating healthy corner stores as well as the neighborhood pedestrian environment surrounding the stores, which is particularly relevant to corner stores and individuals without private transportation options. Additionally, the observation that participants were not generally aware of the Camden Healthy Corner Store Network, though they recognized many of its features as positive, may reveal a need for new outreach opportunities between stakeholders and community residents. Though the possible long-term impacts of this pilot investigation remain to be seen, the continuing dialog between *Our Voice* researchers and TFT staff will allow the effectiveness and sustainability of this type of community engagement to be assessed in the future. The empowerment and activation potential of community-engaged research models like Photovoice and *Our Voice* also potentially enhance the prospect of continued participant engagement (26, 27, 31).

With the proliferation of healthy food access programs in underserved communities across the United States, it is important to ensure that the means of providing *physical* access is appropriate for the target population(s). It is often the case that funders of community-based public health initiatives desire that the perspectives of potential users or beneficiaries are included in program designs (as in Camden’s Healthy Corner Store Network). However, these data are often gathered through structured methods that preclude deeper participant engagement. By using a process that was derived from Photovoice methodologies, this pilot study was able to collect objective and perceived information about community members’ experiences with the corner store intervention, and respond to calls among public health experts for better community engagement in identifying and prioritizing neighborhood environment issues (32, 33).

Researchers and practitioners have encouraged a broad approach for the future of corner store interventions, including improving healthy food availability, offering nutrition education to store customers, and promoting healthy products (11, 34). This pilot demonstrated the viability of directly including community members in gathering and interpreting data to evaluate a corner store program, and make recommendations for its future. In Camden, the results of this study show how the Healthy Corner Store Network is making a positive contribution to the everyday lives of this participant population. Furthermore, it created opportunities to collaboratively modify existing healthy food access programs in ways that better suit the needs of individuals living in this low food access area and that value their local knowledge.

### Limitations

This study has several limitations. First, as a pilot study, a small convenience sample was used, both in terms of participants and corner stores, potentially overlooking harder-to-reach populations and settings. The purposive selection of Stores A and B, while pragmatic and necessary for a small pilot study, inherently limited participants’ ability to comment on the broader healthy corner store program; future assessments should consider having participants evaluate additional, randomly selected stores. Favorability bias may also have influenced participants’ neighborhood assessments and survey responses. Future research in Camden with a larger participant population over multiple time points may help mitigate some of these challenges, and provide a check on the validity of these findings.

### Conclusion

This iteration of *Our Voice* research in healthy corner stores describes a process for researchers and practitioners to formally and directly incorporate relatively unfiltered community perspectives into similar formative evaluations. This pilot study also demonstrates an example of community engagement that empowers participants to collect, analyze, and advocate with their own data, expanding on traditional models of citizen science in a way that is flexible, low-barrier, and action-oriented. Finally, as a multisectoral collaboration between an academic research institution (which provided assessment tools and processes), a nonprofit organization (which manages the healthy corner store program and implemented the assessment), and philanthropic partner (which funds the healthy corner store program and which desired additional community engagement), this study provides a unique evaluation process that could be replicated in other healthy corner store and similarly situated public health interventions.

## Ethics Statement

This study was carried out in accordance with the recommendations of the Institutional Review Board at Stanford University with written informed consent from all subjects. All subjects gave written informed consent in accordance with the Declaration of Helsinki. The protocol was approved by the Institutional Review Board at Stanford University.

## Author Contributions

All authors contributed to the conceptualization of the research questions and design. AR, FS, and TM recruited participants, assisted with data collection, and facilitated participant meetings. BC prepared the manuscript and figures. All authors reviewed and provided comments on the manuscript.

## Conflict of Interest Statement

During the study and preparation of the manuscript, AR, FS, and TM were employed by The Food Trust, and helped manage the Camden Healthy Corner Store Initiative.

## Supplementary Material

Figure S1 Map of Camden, New Jersey, highlighting the North Camden neighborhood where the study took place [map generated in Leaflet (35); Map tiles by Stamen Design CC BY 3.0; Map data© OpenStreetMap; Imagery from GIS Research Group© University of Heidelberg].
