# Supplementary figures and images for: Leveraging Citizen Science for Healthier Food Environments: A Pilot Study to Evaluate Corner Stores in Camden, New Jersey
Source: Front Public Health. 2018 Mar 26;6:89. doi: 10.3389/fpubh.2018.00089 (PMC5879453; doi:10.3389/fpubh.2018.00089)

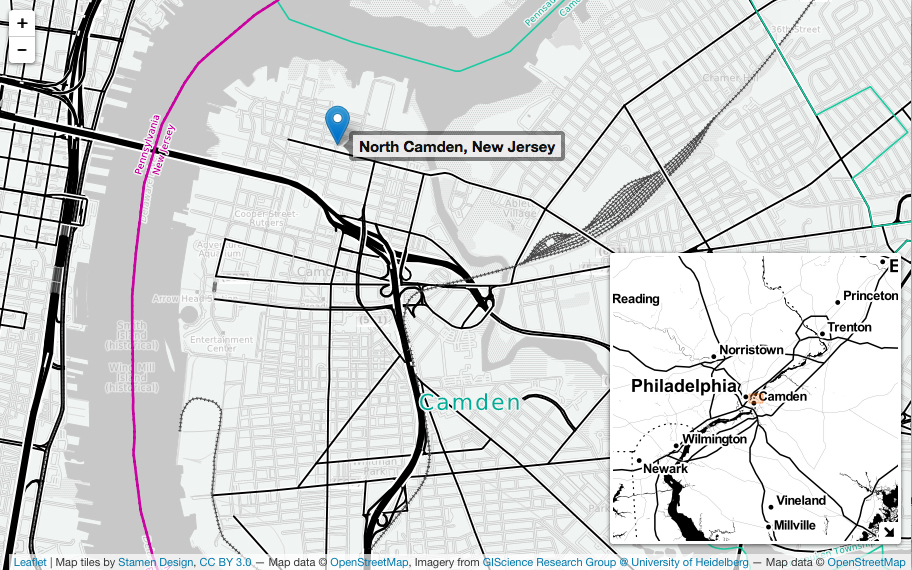

Supplement: Figure S1 — Map of Camden, New Jersey, highlighting the North Camden neighborhood where the study took place [map generated in Leaflet (35); Map tiles by Stamen Design CC BY 3.0; Map data© OpenStreetMap; Imagery from GIS Research Group© University of Heidelberg]. [file Image_1.jpeg]
